# N-Acetylcysteine Ameliorates Loss of the Electroretinogram b-wave in a Bardet-Biedl Syndrome Type 10 Mouse Model

**DOI:** 10.33696/Neurol.6.108

**Published:** 2025

**Authors:** Tyler J. Rankin, Sara Mayer, Joseph G. Laird, Brianna Lobeck, Emily Kalmanek, Arlene V. Drack

**Affiliations:** 1 Department of Ophthalmology and Visual Sciences, University of Iowa, Iowa City, IA, USA; 2 Interdisciplinary Graduate Program in Genetics, University of Iowa, Iowa City, IA, USA; 3 Department of Biochemistry and Molecular Biology, University of Iowa, Iowa City, IA, USA; 4 Department of Pediatrics, University of Iowa, Iowa City, IA, USA

**Keywords:** N-Acetylcysteine, Electroretinogram, b-wave, Immunohistochemistry, Synapse, Optical coherence tomography, Outer nuclear layer

## Abstract

Bardet-Biedl Syndrome (BBS) is a rare autosomal recessive disorder characterized by retinal degeneration leading to blindness. This study investigates the therapeutic efficacy of N-Acetylcysteine (NAC), an oxygen free radical scavenger, in ameliorating retinal degeneration associated with BBS using a murine model of BBS10. BBS is caused by mutations in BBS genes, the protein products of which are involved in ciliary function; mutant or absent BBS10 protein disrupts the assembly of the BBSome protein complex, disturbing ciliary trafficking and leading to photoreceptor cell dysfunction and death. Photoreceptor function can be assessed using the electroretinogram (ERG), and anatomy can be assessed using optical coherence tomography (OCT) and histology to demonstrate progressive degeneration over time. This study utilizes *Bbs10*^−/−^ mice to assess the effect of NAC supplementation on retinal degeneration.

Results reveal that NAC supplementation ameliorates the progressive degeneration of the retinal outer nuclear layer (ONL) on OCT and mitigates the loss-of-b-wave ERG phenotype observed in *Bbs10*^−/−^ mice. The ERG b-wave is generated by retinal bipolar cells after synapsing with photoreceptors which have been hyperpolarized by light exposure. Reducing the loss-of-b-wave phenotype may indicate improved synaptic function. Synaptic staining demonstrates a correlation between the absence of an electropositive b-wave and mislocalized presynaptic terminals, highlighting the significance of synaptic integrity for retinal function. These findings suggest NAC as a promising therapeutic intervention for managing BBS10-related retinal degeneration.

## Introduction

Bardet-Biedl Syndrome (BBS) is a rare, autosomal recessive disorder [1]. The syndrome can result from mutations in any of 29+ BBS genes, most of which are involved in the function of cilia, which play a crucial role in cellular signaling and sensory perception [2,3]. Individuals with BBS commonly exhibit features such as obesity, vision impairment, kidney dysfunction, polydactyly, and developmental or behavioral issues [4–6]. The clinical presentation can vary widely among affected individuals, contributing to the complexity of BBS diagnosis and management.

**Figure 1.**
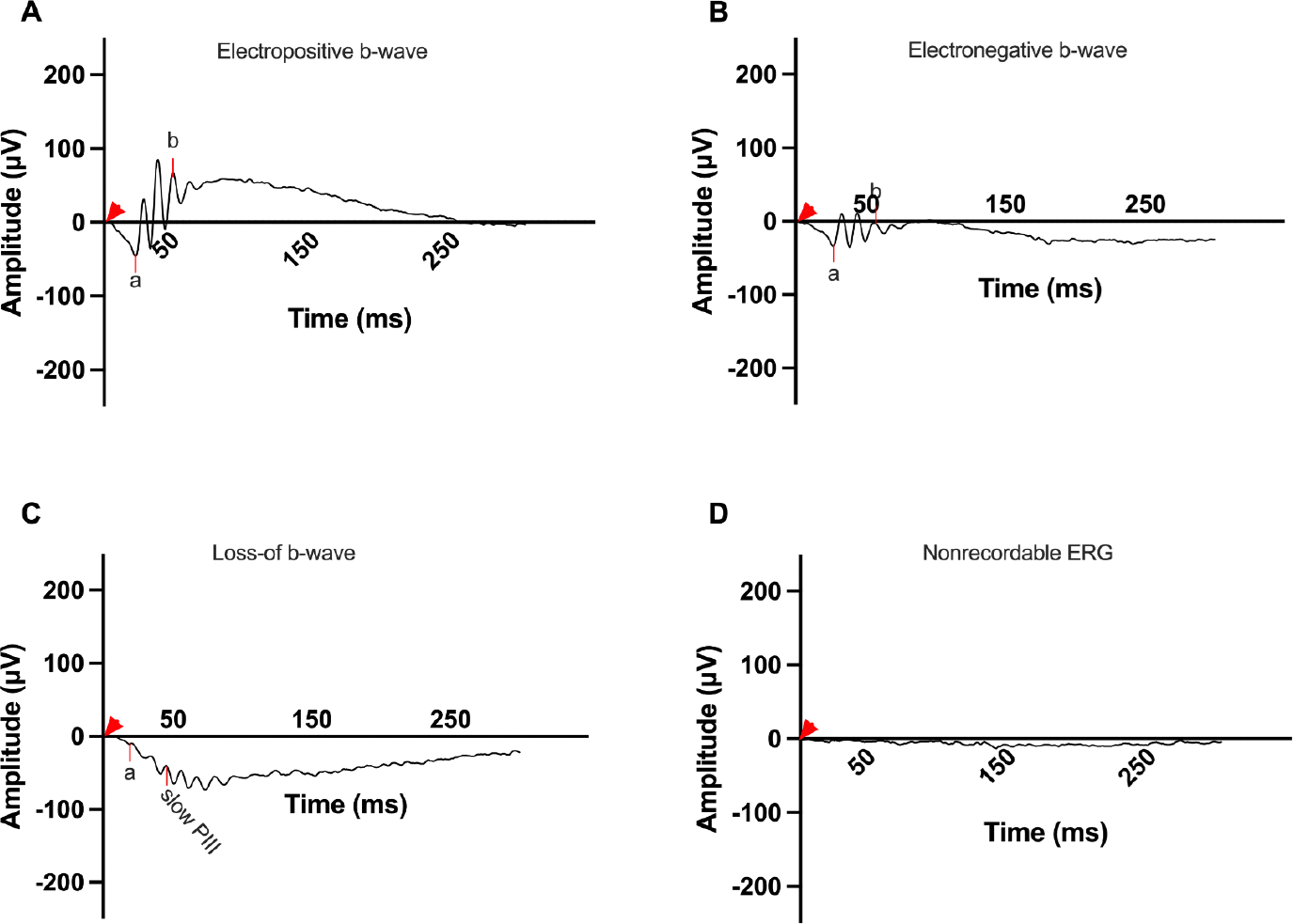
Representative dark adapted 3.0 cd•s/m^2^ ERG b-wave phenotypes in the BBS10 mouse model at around 4 months of age. This figure showcases the range of ERG phenotypes observed in the BBS10 mouse model at around 4 months of age, highlighting the variability in retinal responses among different mice. The a- and b- waves, which reflect photoreceptor and bipolar cell function respectively, are marked. The red arrowhead marks the time the flash was elicited (0 ms). **A:** Electropositive phenotype: This waveform features a pronounced a-wave followed by a slightly higher amplitude b-wave, indicating relatively preserved photoreceptor and bipolar cell function. The phenotype represents a less severe manifestation of retinal degeneration. **B:** Electronegative phenotype: This ERG shows a robust a-wave, indicative of functional photoreceptors. However, the b-wave does not rise above baseline, suggesting significant impairment in bipolar cell activity and synaptic transmission. **C:** Loss-of-b-wave phenotype: This waveform exhibits an a-wave with no subsequent positive b-wave, reflecting severe disruption in the synaptic transmission from photoreceptors to bipolar cells. The presence of a slow electronegative waveform following the a-wave suggests unmasking of the slow PIII wave generated by Mueller cells, usually not seen due to the photoreceptor and bipolar responses. The visible slow PIII indicates considerable retinal dysfunction. **D:** Flat/Nonrecordable phenotype: This ERG is characterized by a flat response with nonrecordable a- and b- waves indicative of severe or complete loss of both photoreceptors and bipolar cell function.

The BBSome, a protein complex involved in ciliary function, is affected in many types of Bardet-Biedl Syndrome. The BBSome is a protein complex composed of BBS1, BBS2, BBS4, BBS5, BBS7, BBS8, and BBS18 [7]. The chaperonin-like complex is composed of BBS6, BBS10, and BBS12 and is important in formation of the BBSome [8]. Mutations in the BBS10 gene generate a mutant chaperonin-like protein that disrupts the assembly and function of the chaperonin-like complex, resulting in improper assembly of the BBSome and causing irregular ciliary transport and signaling pathways [8–10]. The BBSome plays a crucial role in maintaining cellular homeostasis, and its dysfunction contributes to the diverse phenotypes observed in BBS [11]. Vision loss is a prominent and debilitating aspect of BBS10 [12]. Vision is initiated by the hyperpolarization of rods and cones in response to a light stimulus. On the electroretinogram (ERG), this is recorded as the a-wave [13]. This signal is then transmitted through a synaptic region between the photoreceptors and bipolar cells causing depolarization, which is recorded as the b-wave [14]. Vision loss in BBS10 is caused by the dysfunction and deterioration of photoreceptor cells in the retina, due to the improper function of the BBSome [9–12]. The b-wave in an ERG is measured from the trough of the a-wave to the highest peak of the bipolar response b-wave [15]. Four different ERG b-wave phenotypes have been reported for the dark-adapted bright flash with varying nomenclature; in this paper we are using the nomenclature: electropositive, electronegative, loss-of-b-wave/slow PIII, and flat/nonrecordable (Figure 1). Electropositive b-waves surpass baseline positively, while electronegative ones have positive amplitude from the trough of the a-wave but less recovery and do not surpass baseline. The loss-of-b-wave/slow PIII phenotype shows a negative a-wave deflection without a positive counterpart, with the recording after the initial depolarization dropping below the a-wave. The PIII wave is a slow electronegative wave that reflects Müller cell activity [16]. This waveform is not detectable in the presence of the b-wave, due to the large depolarization of bipolar cells which masks Müller cell activity. Flat/nonrecordable b-waves lack distinguishable waveforms and demonstrate only background electrical noise around the baseline (Figure 1).

The retina is a metabolically active tissue with high levels of energy production and consumption and abundant mitochondria that need to accommodate for the high energy demands [17]. Mitochondrial oxidative stress is a major consequence of retinal dystrophies [18]. In retinitis pigmentosa (RP), another retinal abnormality with similar phenotypes to BBS, patients also display reduced levels of glutathione (GSH) in their vitreous and aqueous humor and increased reactive oxygen species [19,20]. The retinal degeneration in individuals with BBS has been linked with oxidative stress [18]. Thus, it is hypothesized to be a contributing factor to disease progression. Patients with BBS were found to have significantly higher levels of mitochondrial fluorescent flavoproteins in the retina which is associated with increased oxidative stress [18]. BBS10^−/−^ kidney-epithelial cells showed increased rates of proliferation and ATP production as well as increased expression of multiple metabolic enzymes such as PDK1, LDH1 and GLUT1 [21]. These results point toward metabolic dysfunction in cells, which is associated with oxidative stress and can contribute to neurodegeneration and retinal degeneration [22]. The antioxidant, NAC, has successfully been used in both preclinical and clinical trials to reduce oxidative damage and increase cone function/survival in humans, preserving photoreceptors and retinal function [23]. NAC functions as an antioxidant primarily by replenishing cellular levels of glutathione. NAC operates at the molecular level as a precursor to cysteine, where it supports the synthesis of glutathione, a key intracellular antioxidant [24]. Glutathione plays a crucial role in neutralizing reactive oxygen species (ROS) and protecting cells from oxidative damage [25].

Since NAC has been proven to be effective in other retinal dystrophies, we performed a preclinical trial with control and *Bbs10*^−/−^ mice supplemented with NAC. In this study, mice received NAC supplemented with their drinking water from birth until four months of age. We evaluated retinal electrical function and analyzed the integrity of retinal layers and presynaptic terminals over time. A unique synapse phenotype was noted as part of the progressive retinal degeneration in *Bbs10*^−/−^ mice; this phenotype was ameliorated by NAC supplementation. Our hypothesis is that BBS10 dysfunction leads to retinal degeneration and synaptic dysfunction, which may be ameliorated by reducing oxidative stress using NAC.

## Methods

### Animal husbandry

This study was performed in strict accordance with the recommendations in the Guide for the Care and Use of Laboratory Animals of the National Institutes of Health. All the animals were handled according to approved Institutional Animal Care and Use Committee (IACUC) protocol #4031421 of the University of Iowa. The *Bbs10* mouse model was acquired from the KOMP2 Center at the Jackson Laboratory, and it is now distributed through the Mutant Mouse Resource and Research Centers (MMRRC); a more detailed description of how the mouse was generated and characterized was previously reported [26]. This mouse was initially on a C57BL/NJ background; however, it was backcrossed onto a 129/SvJ background in the lab of Val Sheffield; this back-crossed model was generously shared with our laboratory.

In this study, *Bbs10*^−/−^ mice were orally treated with NAC. Mice were provided with water bottles containing water only or NAC dissolved in water at a concentration of 7 mg/mL and pH of 4. Breeder pairs were established and were monitored daily for new pups. Prior to giving birth, the breeder female was housed in a cage containing a water bottle with water only. In the day after delivering pups, she was given either water only or water with NAC, depending on the treatment group the pups were assigned. Those given NAC were retained on NAC until all of the pups were weaned. When the pups were weaned, they were placed in their own permanent cage with their own water bottle. Weaned mice were given water bottles that matched the treatments that their mother had experienced during nursing.

Both male and female mice were used in this study. Animals were generated by crossing *Bbs10*^*+/−*^ males with females that were *Bbs10*^+/−^ or *Bbs10*^*−/−*^. The reason for this breeding scheme is that *Bbs10*^−/−^ males are sterile due to lack of functional cilia which are required for motile sperm. *Bbs10*^*−/−*^ females are fertile, so we used both *Bbs10*^−/−^ and *Bbs10*^+/−^ to achieve *Bbs10*^−/−^ mice required for this experiment. Methods of euthanasia used were carbon dioxide inhalation followed by cervical dislocation. Humane endpoints were strictly observed, and every effort was made to minimize suffering.

### Genotyping

Genotyping was performed using mouse tail snips collected at P14. DNA was extracted, and *Bbs10* was amplified using wildtype and knockout forward primers and the same reverse primer shown in Table 1.

### Optical Coherence Tomography (OCT)

OCT can be utilized to visualize the cellular layers of the retina in a living mouse. OCT was performed using the Bioptigen OCT machine with a small rodent lens (Research Triangle Park, NC). Mice were anesthetized with a mixture of ketamine (87.5 mg/kg) and xylazine (2.551 mg/kg), and pupils were dilated with tropicamide 1%. The eyes were then lubricated with Refresh Liquigel carboxymethylcellulose sodium eye drops and the mice were placed in such a way that the noncontact lens of the OCT machinery could be positioned near the eye. The lens was drawn close to the cornea until the image of the retina was visible on the computer screen. The retina was oriented on the screen so that the optic nerve was centered in both the horizontal and vertical positions, indicating the image was of the center of the retina. OCT images are performed using a circular volumetric acquisition scan with a diameter of 1.40 mm. The OCT images are aligned horizontally and represent a slice in the frontal plane of the mouse. All images are a central OCT scan from temporal to nasal sides of the mouse eye. For all images, measurements of the outer nuclear layer (ONL) were taken from 300 μm on either side of the optic nerve, using the in-software calipers provided by Bioptigen.

### Electroretinogram (ERG)

ERG is used to quantify the electrical response of the retina to a light stimulus. A light flashed on a photoreceptor will induce an electrical response chain reaction starting in the photoreceptor and amplified by the bipolar cells and then the ganglion cells. This signal will be transferred to the brain and be interpreted by the occipital lobe communicating with other centers of the brain as sight. This electrical response of the cells in the retina can be recorded with an ERG. Full-field electroretinograms (ERGs) were conducted using the Celeris Diagnosys system (Diagnosys LLC, Lowell, MA). The mice were dark adapted overnight before ERG was performed. Mice were anesthetized with a mixture of ketamine (87.5 mg/kg) and xylazine (2.5 mg/kg). The mice received 0.1 mL of the mixture per 20 g body weight. ERGs were recorded from the corneal surface of each eye, after the pupils were dilated with 1% tropicamide, using Diagnosys Celeris touch/touch stimulator electrodes. Gonak gel (Akorn, Inc., Lake Forest, IL) was placed on the cornea of each eye before the electrode was positioned. Light flashes were produced by the touch stimulator electrodes. Dim red light was used to illuminate the room until dark-adapted testing was completed. A modified International Society for Clinical Electrophysiology of Vision (ISCEV) protocol was used [27]. A dim flash of 0.01 cd•s/m2 was first used to stimulate rods, followed by a bright flash at 3.0 cd•s/m2 to measure the standard combined response (SCR) of the rods and cones. Mice were then light adapted for 10 minutes, after which they were tested with a bright flash at 3.0 cd•s/m2 followed by a flickering light of the same intensity at 5Hz. For most measurements, 15 sweeps were taken per condition; for the 5 Hz flicker, 20 sweeps were taken. ERG results were analyzed following data collection, using the Diagnosys software to eliminate sweeps that show interference such as mouse movements or ambient electric signal. Any test in which more than five sweeps were needed to eliminate interference was considered null and it was repeated for cleaner results. The light-adapted protocol tests cones, especially the 5 Hz flicker, which in mice elicits a response from only cones.

### Histology

Eyecups from the posterior region were dissected in 1xPBS pH 7.4, then fixed in 4% paraformaldehyde for 15 minutes at 4°C. They were subsequently cryoprotected in 30% sucrose solution at 4°C overnight and frozen in O.C.T. compound (Tissue-Tek, Electron Microscopy Sciences, Hatfield, PA). Radial sections were cut and placed on electrostatically charged glass slides. Blocking buffer containing 10% normal goat serum and 0.5% Triton X-100 in PBS pH 7.4 was applied. Primary antibodies diluted 1:250 – 1:500 in blocking buffer were allowed to incubate on retinal sections overnight at 4°C, while secondary antibodies diluted 1:500 in blocking buffer were allowed to incubate on retinal sections for 1–3 hours at room temperature. Imaging was conducted using a THUNDER Imager Leica DM6B microscope equipped with a Leica DFC9000 GT camera and computational clearing of z-stacks was carried out using LASx software generously loaned by the laboratory of Sheila Baker.

### Antibodies

Nuclei were stained with Hoechst 33342 at a 1:1000 dilution. Primary antibodies used were anti-CtBP2/RIBEYE antibody (BD Transduction Laboratories #612044), anti-PSD95 Recombinant Rabbit Monoclonal antibody (SR38-09) (Life Technologies) both used at a 1:500 dilution and anti-PKCα antibody (Invitrogen #MA5-32106) at 1:250. Secondary antibodies used were Anti-mouse conjugated to Alexa 594 and Anti-rabbit conjugated to Alexa 488 at 1:500 dilution.

### Presynaptic terminal mislocalization count

A total of two cryosections were imaged from each of three different retinal locations (dorsal, central, and ventral) per ERG phenotype. These images were obtained to avoid the optic nerve and ensure the best morphology of the presynaptic terminals. Each section was imaged to capture a representative location of the retina. The images were processed using Photoshop to outline the outer nuclear layer (ONL) by employing the magic lasso tool to delineate the area stained by Hoechst. Two masked examiners were then provided with both the processed ONL-only images and the original unprocessed images to count the number of presynaptic terminals in the ONL. For a presynaptic terminal to be considered, it had to show a PSD-95 “cup” adjacent to CtBP2 (RIBEYE) staining. Each retinal image covered a width of 209 μm, with a single image area calculated to be 43,681 μm^2^. Given that images from three distinct locations were analyzed per retina, the total area for one retina was 131,043 μm^2^ (43,681 μm^2^ × 3). With two different retinal sections analyzed, the final total area observed was 262,086 μm^2^ (131,043 μm^2^ × 2). This methodology provides a thorough evaluation of mislocalized presynaptic terminals by covering multiple retinal regions and maintaining objectivity through masked examination.

### Statistical analysis

To assess the effects of NAC treatment and water-only on retinal outcomes, we conducted experiments using two cohorts of mice. One cohort contained *Bbs10*^+/−^ and *Bbs10*^−/−^ mice reared on water only and investigated at three months of age for ERG, ONL thickness and pre- and post- synaptic terminal imaging. The other cohort contained *Bbs10*^+/−^ and *Bbs10*^−/−^ groups of mice treated since birth with either water or NAC and analyzed at four months of age for ERG, ONL, OPL, and INL thickness on OCT.

### Three-month-old mice reared on water

*Bbs10*^−/−^ *and Bbs10*^+/−^ mice had ERG performed weekly starting at three-months of age in order to identify mice at each of the stages of ERG phenotype of interest. One mouse (two eyes) was identified with each of the three ERG phenotypes and immediately after the ERG was performed the mice were sacrificed and the eyes prepared for histologic examination.

Following histological staining, a one-way ANOVA with Tukey’s post-hoc analysis was conducted on three-month-old water-reared mice to analyze ONL thickness and presynaptic terminal mislocalization. Control heterozygous mice had significantly thicker ONL compared to *Bbs10*^−/−^ with either b-wave, electronegative, or loss-of-b-wave (p<0.0001). No differences were observed among the latter three groups.

On quantification of presynaptic terminals, the same dataset of three-month-old water-treated mice was used. Mice that exhibited loss-of-b-wave had significantly more mislocalized presynaptic terminals compared to control mice (p=0.0077), with the loss-of-b-wave group showing the highest counts of mislocalized terminals (p=0.0065). Mice possessing a b-wave had fewer mislocalizations than loss-of-b-wave mice (p=0.0492), suggesting presynaptic terminal mislocalization is related to the loss-of b-wave.

### Four-month-old mice reared on water vs reared on water containing NAC

Thirty-three mice, separated into 4 cohorts as described in Methods and below, were reared on water only vs water containing NAC. For these mice ERG and OCT were performed at four-months of age, followed by sacrifice.

A one-way ANOVA with Bonferroni post-hoc analysis was used to compare ONL, OPL, and INL thickness between the four groups of mice treated since birth with either water or NAC and investigated at four months of age. For these analyses, six water-treated *Bbs10*^+/−^, nine NAC-treated *Bbs10*^+/−^, nine water-treated *Bbs10*^−/−^, and nine NAC-treated *Bbs10*^−/−^ were used. Measurements were taken from both eyes of each mouse, with each eye used as a separate data point. ONL thickness was significantly thinner in water-treated *Bbs10*^−/−^ mice compared to NAC-treated *Bbs10*^−/−^ mice (p=0.0002), indicating that NAC preserves ONL thickness. By contrast, no significant differences were observed for the OPL between water- and NAC-treated *Bbs10*^−/−^ mice. However, differences were observed between water-treated *Bbs10*^−/−^ and NAC-treated *Bbs10*^+/−^ mice (p=0.0041). INL thickness showed minimal changes among the groups of mice, with the exception being a difference between water-treated *Bbs10*^+/−^ and NAC-treated *Bbs10*^−/−^ mice (p=0.0069). Differences in INL thickness were not different between water- and NAC-treated *Bbs10*^−/−^ mice.

A Welch’s t-test was performed for a- and b-wave amplitude analysis using the same four groups of mice treated since birth with either water or NAC and investigated at four months of age. The number of mice in each group was the same as above. No significant difference was observed in a-wave amplitudes between water- and NAC-treated *Bbs10*^−/−^ mice (p=0.9378), indicating no effect of NAC on a-wave amplitudes. However, b-wave amplitudes were significantly lower in water-treated *Bbs10*^−/−^ mice compared to NAC-treated mice (p=0.0029), demonstrating NAC treatment improves b-wave amplitudes. Following this, a Fisher’s exact test was used to determine whether the presence of b-waves was different between the groups. This analysis showed that a significantly lower proportion of water-treated *Bbs10*^−/−^ mice retained b-waves compared to NAC-treated mice (p=0.0036). Thus, NAC treatment preserves b-waves at four months of age.

### The ROUT method (Q = 1%) detected no outliers in both datasets, confirming reliability

In summary, NAC supplementation from birth significantly improved ONL thickness and b-wave amplitudes in *Bbs10*^−/−^ mice compared to water-treated *Bbs10*^−/−^ mice. Additionally, a significant proportion of NAC-treated *Bbs10*^−/−^ mice retained a b-wave, unlike the water-treated group. These findings demonstrate the beneficial effects of NAC treatment on retinal degeneration in *Bbs10*^−/−^ mice.

## Results

In this study, we investigated the natural history of ERG b-wave evolution in *Bbs10*^−/−^ mice, the effects of NAC treatment on retinal structure and function, and the synaptic changes associated with different ERG phenotypes. Our key findings include the progressive loss of b-wave responses in *Bbs10*^−/−^ mice, the preservation of outer nuclear layer (ONL) thickness with NAC treatment, and longer preservation of the b-wave in *Bbs10*^−/−^ mice reared on NAC.

### Natural history of ERG b-wave in the *Bbs10*^−/−^ mouse model

**Figure 2.**
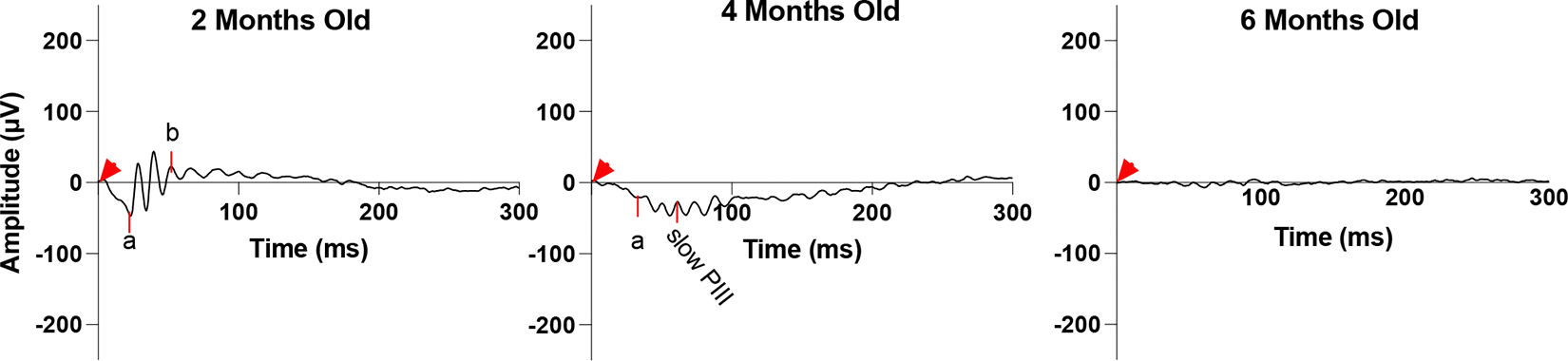
Progressive degeneration in dark adapted 3.0 cd•s/m^2^ ERG b-wave in a representative *Bbs10*^−/−^ mouse. This figure illustrates the progressive decline in retinal function over time in a representative *Bbs10*^−/−^ mouse, as evidenced by changes in the ERG b-wave responses, with the red arrowhead representing the time the flash was elicited (0 ms). At 2 months old, the initial ERG shows a robust a-wave, reflecting persistent photoreceptor activity, followed by an electropositive b-wave, indicating functional bipolar cells and synaptic transmission. This represents the early stage of retinal degeneration. At 4 months old, the ERG demonstrates a transition into an absent b-wave phenotype. The a-wave remains present, signifying continued, though declining photoreceptor function. However, the absence of the b-wave suggests a significant loss of synaptic activity and bipolar cell function. At 6 months old, the ERG is flat and nonrecordable, indicative of severe retinal degeneration. Both a- and b- waves are absent, reflecting a profound loss of photoreceptor and bipolar cell function, and marking the advanced stage of retinal degeneration. This figure highlights the temporal progression of retinal degeneration in *Bbs10*^−/−^ mice, demonstrating how the disease evolves from relatively mild to severe impairment within a span of several months.

Figure 2 illustrates the evolution of ERG b-waves in *Bbs10*^−/−^ mice over a six-month period. At 2 months of age, *Bbs10*^−/−^ mice show a reduced b-wave amplitude compared to wild-type (WT) mice, although it is still present. By 4 months, two distinct ERG phenotypes emerge. In one phenotype, the b-wave amplitude decreases, resulting in an abnormal electronegative ERG response where the b-wave does not exceed the baseline. This indicates impaired but not entirely absent transmission from photoreceptors to bipolar cells. In the other phenotype, termed “loss-of-b-wave”, the b-wave is completely absent following the a-wave. This absence indicates a failure of synaptic transmission from photoreceptors to bipolar cells. The absent b-wave phenotype is accompanied by a continued negative deflection, which is linked to Müller cell activity rather than photoreceptors continuing to generate graded potentials in response to light. This is distinct from the ERG waveform recorded when there is little or no photoreceptor activity, termed a flat or nonrecordable ERG. By 6 months, both photoreceptor and Müller cell responses become undetectable, suggesting that, as the disease progresses, photoreceptors lose their ability to respond to light stimuli, reflecting the neurodegenerative nature of the disease and resulting in no propagation of signal to bipolar or Müller cells. It is reported among neurodegenerative diseases for the axon to lose function first, followed by the neuronal cell body apoptosis, known as “dying back” [28,29]. This could be a potential explanation as to why the *Bbs10*^−/−^ mouse model presents these different ERG phenotypes even as the ONL thickness and nuclei appearance remain consistent.

### Synaptic changes observed by immunohistochemistry

**Figure 3.**
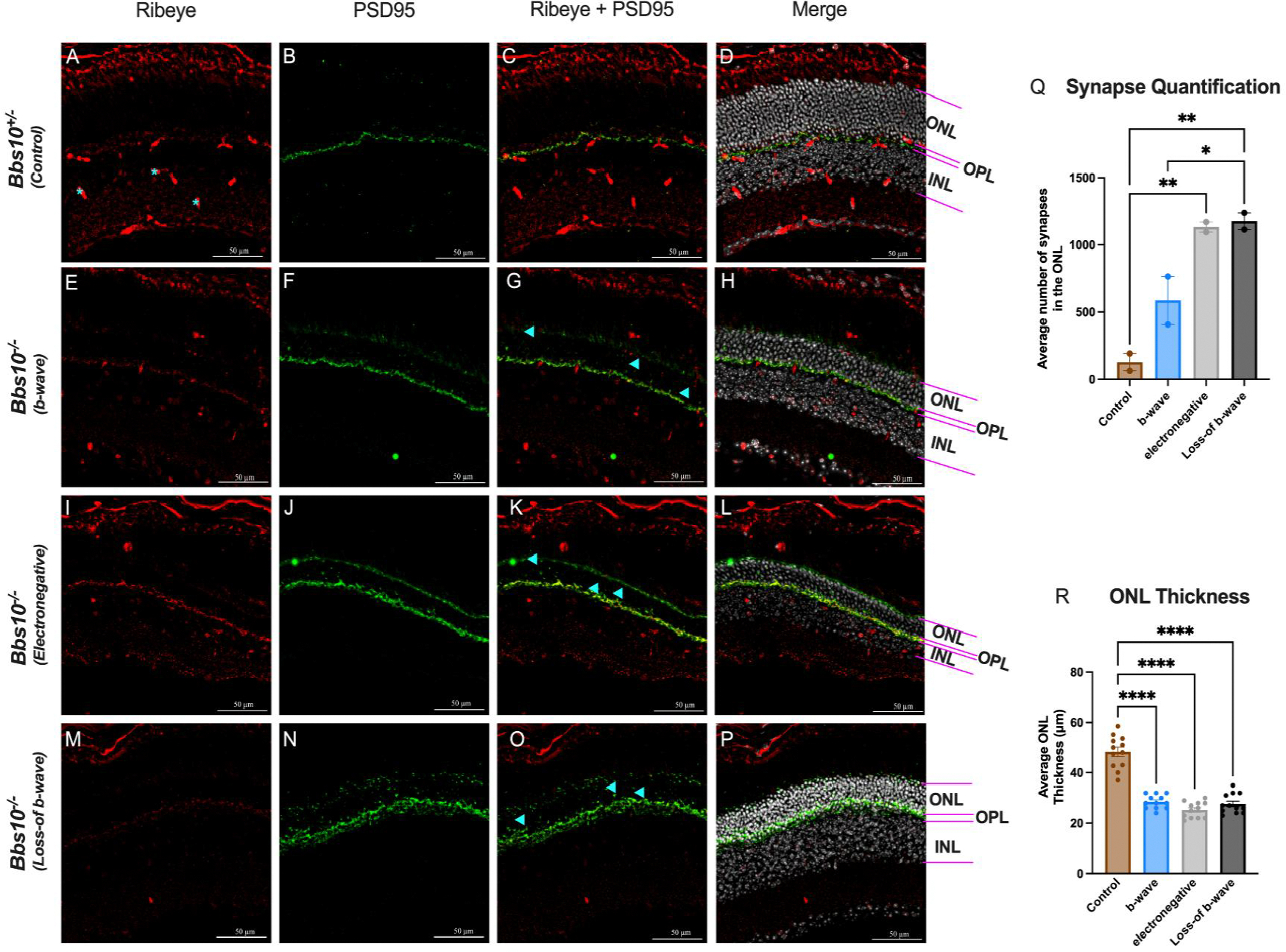
Quantification of presynaptic terminal localization and ONL thickness in retinas of Bbs10^−/−^ mice with different ERG phenotypes. This figure demonstrates the impact of different ERG phenotypes on synapse localization and ONL thickness in Bbs10^+/−^ and Bbs10^−/−^ mice. A few blue asterisks mark ribeye blood vessel staining and not actual presynaptic terminals. Blue arrowheads provide a few examples of what is considered a “mislocalized presynaptic terminal”. **A-P:** Representative images showing the presynaptic markers ribeye (red) and PSD95 (green) staining in retinal sections. The ONL, OPL, and INL are labeled for reference. The panels depict: **A-D:** Control Bbs10^+/−^ with normal ERG, showing presynaptic localization predominantly in the OPL. **E-H:** Bbs10^−/−^ mouse with a low amplitude, positive b-wave ERG, displaying some synaptic mislocalization into the ONL. **I-L:** Bbs10^−/−^ mouse with an electronegative ERG, showing extensive synapse mislocalization into the ONL. **M-P:** *Bbs10*^−/−^ mouse with an absent b-wave ERG, characterized by severe synaptic mislocalization and disrupted retinal layering. **Q:** Quantification of synapse mislocalization in the ONL, showing a statistically significant increase in synapse mislocalization in *Bbs10*^−/−^ compared to control. Among them, those with an electronegative and loss-of- b-wave ERG had significantly more mislocalized presynaptic terminals than those with an electropositive b-wave, with loss-of-b-wave displaying the most mislocalization. **R:** Quantification of ONL thickness, showing that control mice have a statistically thicker ONL, compared to the *Bbs10*^−/−^ mice. No significant difference is ONL thickness were observed among the different *Bbs10*^−/−^ ERG phenotypes. Statistical significance was assessed using an ordinary one-way ANOVA with Tukey’s multiple comparisons test. Significance levels are denoted as ns (non-significant); * (p≤0.05); ** (p≤0.01); *** (p≤0.001); **** (p≤0.0001).

To understand the anatomical basis for the different ERG phenotypes, we performed immunohistochemistry (IHC) on retinas from untreated three-month-old *Bbs10*^−/−^ mice, which exhibit a mix of ERG phenotypes. Using presynaptic markers PSD95 and CtBP2 (ribeye), we observed that in *Bbs10*^−/−^ mice, presynaptic terminals are often misplaced, with some retracting into the ONL. This mislocalization indicates that synaptic terminals are not properly aligned within the outer plexiform layer (OPL) (Figures 3A–3P). Quantitative analysis of presynaptic terminal density in different retinal quadrants revealed that the absent b-wave phenotype has a higher average number of presynaptic terminals in the ONL compared to retinas with a b-wave response. The presynaptic terminal quantification was collected in a blinded manner, using the explained protocol in the methods section. The quantification revealed a statistically greater number of presynaptic terminals in the ONL of the loss-of-b-wave eyes compared to the electropositive b-wave eyes (Figure 3Q). This suggests that the loss of the b-wave phenotype is associated with significant synaptic disorganization and potential misplacement.

Since BBS10 is a degenerative disease, we also quantified the thickness of the ONL to correlate the difference in presynaptic terminals between phenotypes with the number of photoreceptors. The thickness of the ONL was quantified from the same retinal sections used for synaptic quantification (Figure 3R). The average ONL thickness between the knockout *Bbs10* mice used for presynaptic terminal staining was not statistically different, but the control mouse was significantly thicker than all knockouts (Figure 3R). This is indicative of similar levels of retinal degeneration, strengthening our findings and claim that the ERG phenotype is due to synaptic disorganization and potential misplacement and not more severe degeneration.

**Figure 4.**
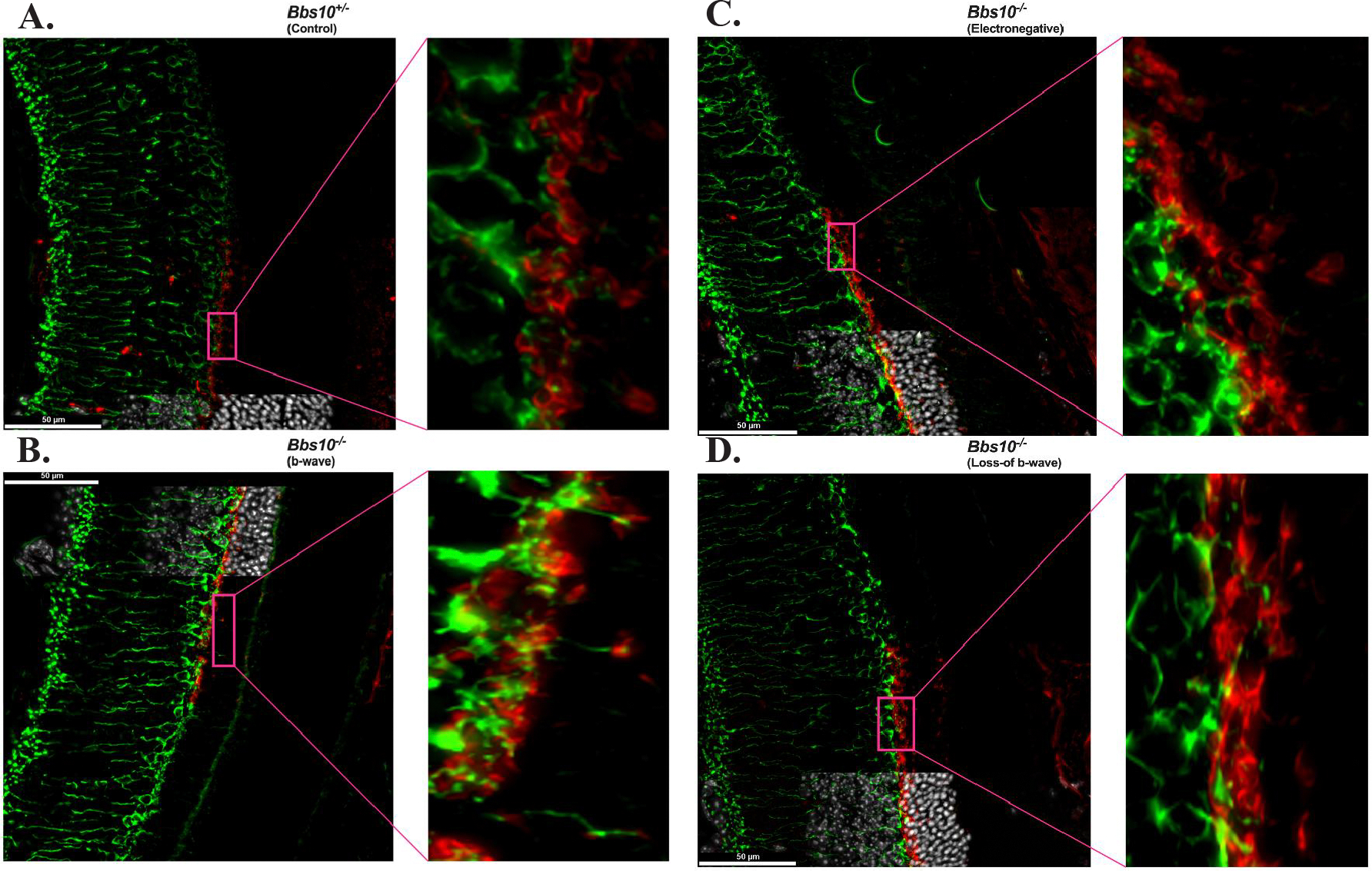
Mislocalization of synaptic markers and loss of synaptic connectivity in the ONL of *Bbs10* mutant mouse retinas. Representative immunofluorescence images of retinal sections with presynaptic marker PSD95 (red) and postsynaptic marker PKCα (green) are shown with Hoechst staining the nuclei from *Bbs10*^*+/−*^ (control) and *Bbs10*^−/−^ mice at different functional states: **A.** control (*Bbs10*^+/−^), synaptic markers are localized to the OPL, with clear connectivity between PSD95 and PKCα. **B.** *Bbs10*^−/−^ with an electropositive b-wave, synaptic markers progressively mislocalize into the ONL, but retain some fully structural pre- and post- synaptic connections as highlighted in the magnified insets (pink call-out box). **C.** *Bbs10*^−/−^ with an electronegative b-wave, synaptic markers become progressively more mislocalized into the ONL, with very few structural pre- and post- synaptic connections as highlighted in the magnified inset (pink call-out box). **D.** *Bbs10*^*−/−*^ with a loss-of b-wave, synaptic markers are highly mislocalized into the ONL, with no full structural pre- and post- synaptic connections as highlighted in the magnified inset (pink call-out box). Scale bar = 50 μm.

To assess synaptic connectivity across four different retinal phenotypes, we performed immunohistochemical staining for the presynaptic marker PSD95 and the postsynaptic marker PKCα. The localization of these markers was evaluated in the OPL and the ONL and these molecular observations were correlated with the ERG data to understand the functional implications of synaptic disruption (Figure 4).

In the control retina (Figure 4A), PSD95 and PKCα exhibited typical synaptic localization. PSD95 was primarily restricted to the OPL, where it formed normal synaptic connections. PKCα was also present in the OPL, co-localizing with PSD95 at postsynaptic sites. The precise synaptic organization observed here corresponds with a normal b-wave on the ERG, reflecting intact synaptic function and signal transmission.

In the eyes with an electropositive b-wave phenotype (Figure 4B), PSD95 was mislocalized into the ONL, indicating a disruption in synaptic organization. Despite this, PKCα was observed in the ONL, where dendritic tips were seen making connections to PSD95 cups, suggesting that complete structural synaptic connections are still being formed, albeit in an altered spatial context. The presence of these PKCα dendritic tips connecting with PSD95 in the ONL likely accounts for the presence of the b-wave on the ERG, indicating that synaptic function was partially maintained, even with the mislocalization of PSD95.

In the electronegative ERG phenotype (Figure 4C), PSD95 mislocalization was even more pronounced, with a greater amount of PSD95 found in the ONL compared to the b-wave phenotype. While PKCα was still present in the ONL, fewer dendritic tips were observed making connections with PSD95, and the formation of synaptic contacts was less frequent and less robust. This reduced ability to form structurally complete synapses likely contributes to the altered ERG response, characterized by a more negative b-wave. The decreased number of PKCα dendritic tips reaching PSD95 cups suggests a further decline in synaptic efficiency.

In the loss-of b-wave phenotype (Figure 4D), PSD95 mislocalization in the ONL was most severe, with large clusters of PSD95 observed far from its normal OPL region. In this phenotype, PKCα was largely confined to the OPL, and dendritic tips from PKCα rarely reached the PSD95 cups in the ONL. The very few connections that were made between PKCα and PSD95 were largely ineffective, corresponding with the absence of the b-wave on the ERG. This lack of synaptic connectivity and signal transduction at the synaptic level explains the complete failure of the b-wave in the ERG.

### NAC treatment

To observe whether NAC might help reduce the burden in retinal degeneration, *Bbs10*^−/−^ were treated with NAC. Mice were provided with water bottles containing either plain water or a solution of NAC at a concentration of 7 mg/mL and pH 4. Breeder pairs were set up and monitored daily for new litters. Before giving birth, the breeder female was housed with a water bottle containing plain water. On the day after delivering the pups, she was given either plain water or water with NAC, depending on the treatment group assigned to the pups. NAC-treated mothers continued to receive the NAC solution until all pups were weaned. Once weaned, the pups were moved to their own cages, each equipped with a water bottle. The water bottles in these cages were matched to the treatment their mother had received during the lactation.

### NAC treatment retains ONL thickness

**Figure 5.**
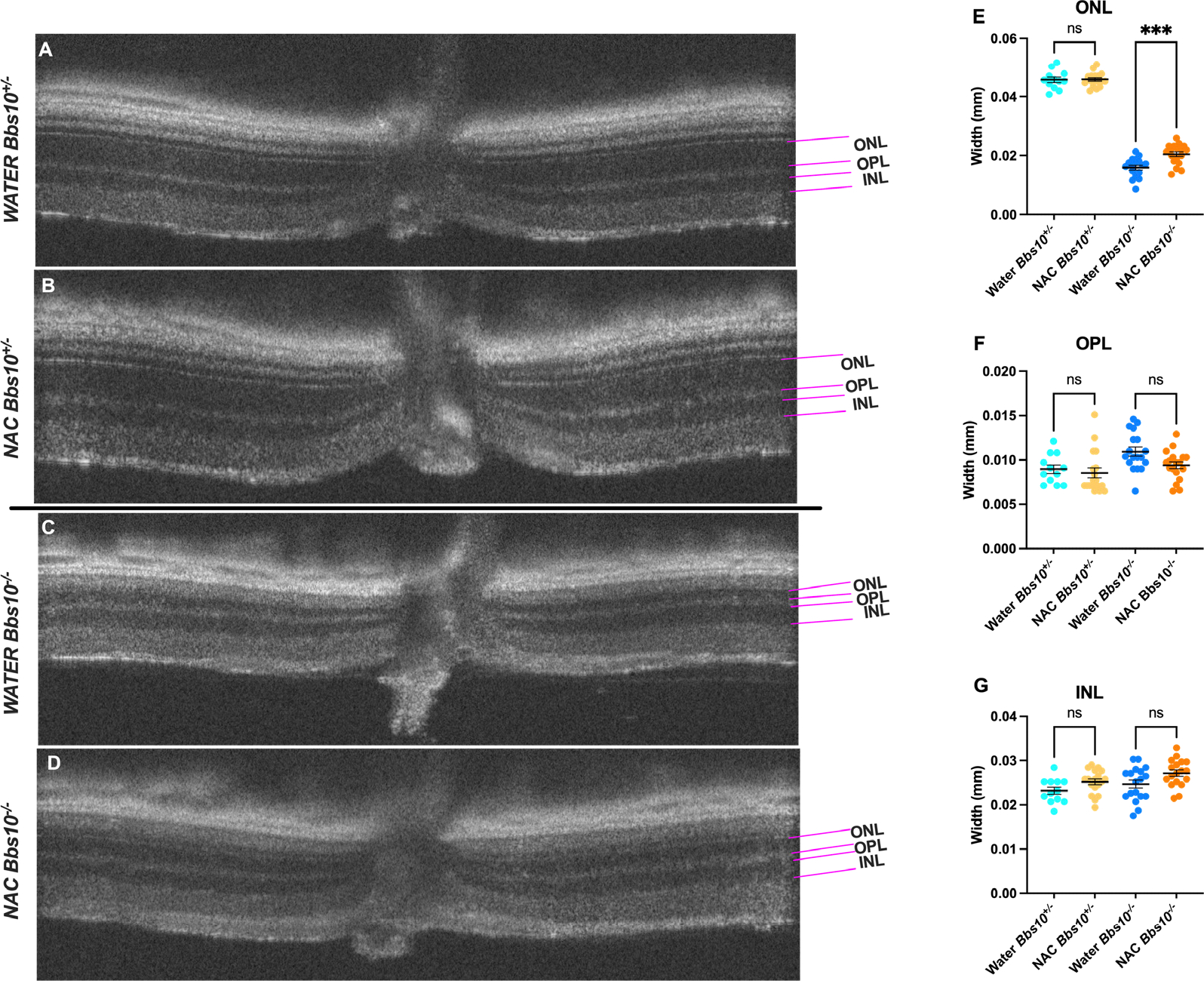
Retinal layer thickness in water-treated and NAC-treated Bbs10^−/−^ mice at 4-months-old. This figure illustrates the differences in retinal layer thickness observed in OCT scans of *Bbs10*^+/−^ and *Bbs10*^−/−^ mice treated with either water or NAC. **A-D:** Representative OCT images show the Outer Nuclear Layer (ONL), Outer Plexiform Layer (OPL), and Inner Nuclear Layer (INL) for each treatment group: **A:** *Bbs10*^+/−^ mouse treated with water. **B:** *Bbs10*^+/−^ mouse treated with NAC. **C:** *Bbs10*^−/−^mouse treated with water. **D:** *Bbs10*^−/−^ mouse treated with NAC. **E:** ONL thickness measurements across all eyes in each treatment group. A significant reduction in ONL thickness is observed in *Bbs10*^−/−^ mice compared to Bbs10^+/−^ mice, with NAC *Bbs10*^−/−^ mice showing a thicker ONL than water *Bbs10*^−/−^. **F:** OPL thickness measurements for each group. The analysis shows no significant differences between water and NAC *Bbs10*^+/−^ or water and NAC *Bbs10*^−/−^ groups. **G:** INL thickness measurements for each group, showing no difference between water and NAC *Bbs10*^+/−^ or water and NAC *Bbs10*^−/−^ groups.

OCT was used to assess retinal structure, specifically the ONL thickness, in *Bbs10*^−/−^ mice treated with either water or NAC (Figure 5). Representative OCT images showed that in *Bbs10*^+/−^ mice, ONL thickness remains consistent over time, regardless of NAC treatment (Figures 5A–5B). In contrast, *Bbs10*^−/−^ mice treated with NAC exhibited a slower decline in ONL thickness compared to those treated with water (Figure 5C–5D). This suggests that NAC treatment has a protective effect on the photoreceptor layer, helping to maintain ONL integrity. The thickness of the outer plexiform layer (OPL) and inner nuclear layer (INL) did not show significant differences between treatment groups, indicating that NAC primarily benefits the photoreceptor and does not affect other retinal layers (Figure 5E–5G). No difference between OPL thickness indicates that we are not seeing a difference in number of synaptic connections, which is why they are of similar thickness, but rather a functional difference which cannot be seen on OCT, but only on ERG.

The data from the two experiments above demonstrate that the preservation of the ONL and the preservation of the b-wave are distinct. At 3 months of age, we observe the different ERG phenotypes despite similar ONL thicknesses across groups. This is consistent with the loss-of b-wave phenotype developing because the synaptic connection between photoreceptors and bipolar cells is lost, not because a critical number of photoreceptors have been lost, which was the alternate hypothesis for why this phenotype evolves. The synaptic function—particularly the wiring between photoreceptors and bipolar cells—may still be compromised even if the ONL is intact, and as a biological process in the evolution of pan-retinal degeneration, it is detected at slightly different ages in untreated mice. For the water raised cohort, we did the initial ERG at 90 days old on three BBS10 KO mice. One mouse had an electropositive b-wave and two had an electronegative b-wave. We sacrificed the electropositive and one electronegative b-wave. We then repeated the ERG on the other electronegative b-wave exactly one-week later, which then developed into the loss-of b-wave.

In the other experiment, in which mice were aged to 4 months, NAC treatment was shown to stabilize synaptic function, thereby preserving the b-wave in at least half of the mice at this age. By 4 months of age, changes in ONL thickness could be detected between NAC treated and untreated knockouts, which correlated with increased prevalence of the b-wave but was not always in the eyes with the thickest ONL. This distinction suggests that the b-wave preservation is not solely dependent on ONL thickness but on the proper synaptic connections and signal transmission between photoreceptors and bipolar cells. The b-wave preservation likely results from NAC stabilizing synapses by improving function of photoreceptors and/or bipolar cells.

### NAC treatment and b-wave amplitudes

**Figure 6.**
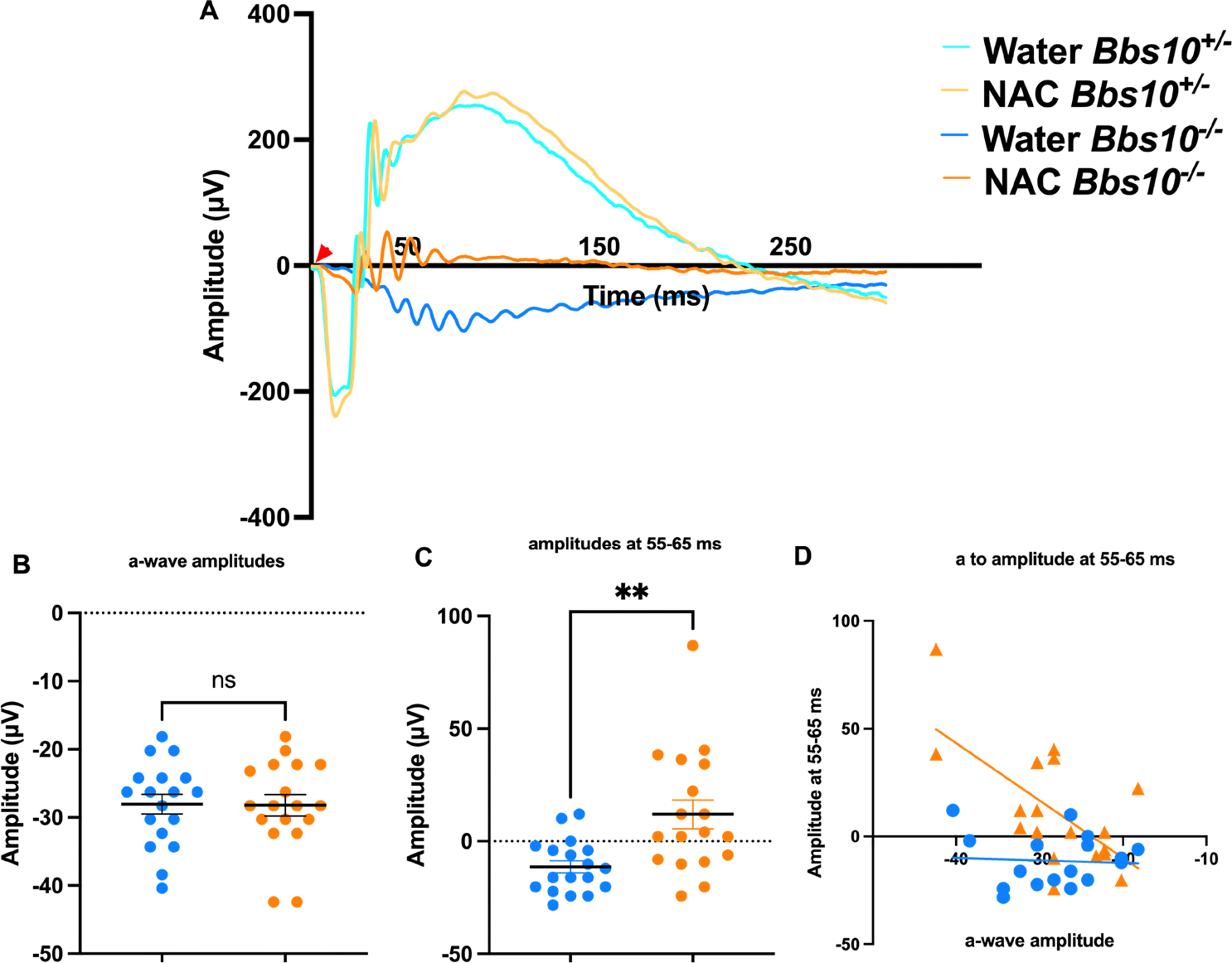
ERG amplitudes in 4-month-old *Bbs10*^−/−^ mice show increased b-wave amplitudes with NAC treatment. This figure demonstrates the impact of NAC treatment on the electroretinography (ERG) responses of 4-month-old *Bbs10*^−/−^ mice, specifically focusing on the standard combined response. The red arrowhead represents when the stimulus flash was elicited (0 ms). **A:** Representative waveforms of the standard combined response from a single eye in each genotype and treatment group. The waveforms illustrate the a-wave and b-wave components of the ERG, with visible differences in the b-wave amplitude between NAC-treated and water-treated *Bbs10*^−/−^ mice. **B:** Quantification of the a-wave amplitudes across all eyes shows no significant difference between NAC-treated and water-treated *Bbs10*^−/−^ mice, indicating that NAC does not significantly affect the initial photoreceptor response. **C:** Quantification of the absent b-wave/slow PIII amplitudes reveals a statistically significant increase in the NAC-treated *Bbs10*^−/−^ mice compared to the water-treated controls (Welch’s t-test, p = 0.0029), suggesting that NAC treatment improves inner retinal function. **D:** A correlation analysis between the a-wave and the absent b-wave/slow PIII amplitudes is shown, using a simple linear regression model. In the water-treated controls, no significant correlation is observed, while NAC treatment reveals a significant positive correlation (p=0.0036), indicating that the improved inner retinal response in NAC-treated mice is related to the initial photoreceptor activity. These findings suggest that NAC treatment enhances the inner retinal response in Bbs10^−/−^ mice, as evidenced by the increased amplitude of the absent b-wave/slow PIII, while the a-wave remains unaffected.

**Figure 7.**
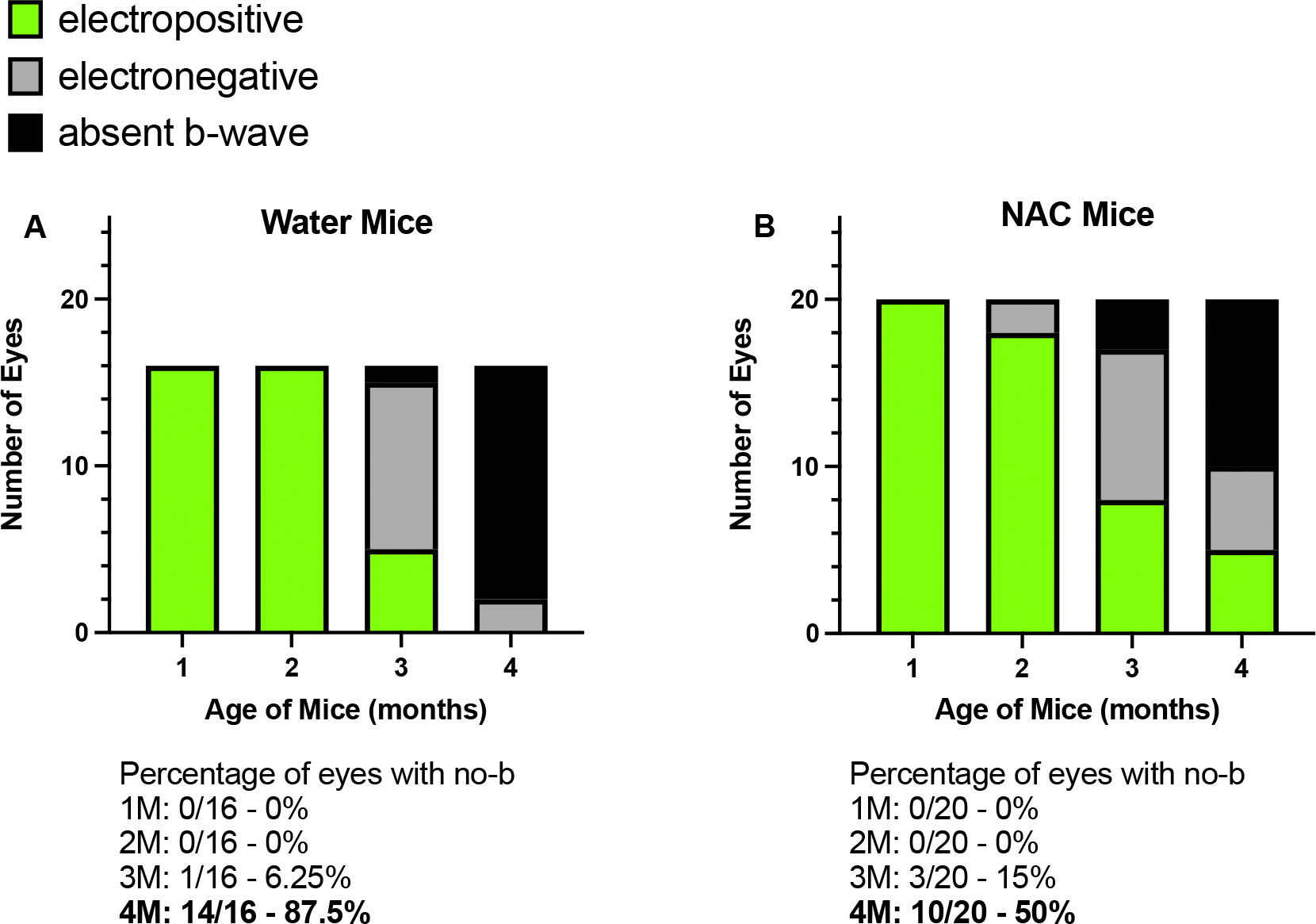
Transformation of SCR b-wave over time in untreated vs. NAC-treated Bbs10^−/−^ mice. This figure illustrates the effect of NAC treatment on the presence of a standard combined response SCR b-wave in 4-month-old *Bbs10*^−/−^ mice, comparing untreated (normal drinking water) and NAC-treated groups. **A:** In the untreated group, *Bbs10*^−/−^ mice that received normal drinking water exhibited a higher percentage of mice with absent b-wave at 4 months old. The graph highlights the significant proportion of these mice that lack the b-wave, indicating impaired inner retinal function. **B:** In the NAC-treated group, *Bbs10*^−/−^ mice that received water supplemented with 7 mg/mL NAC showed a reduced percentage of absent b-wave mice at 4 months old. The data suggests that NAC treatment leads to a notable preservation or restoration of the b-wave, reflecting an improvement in retinal function. This figure underscores the potential therapeutic effect of NAC in reducing the number of *Bbs10*^−/−^ mice with absent b-wave/slow PIII responses, thereby enhancing inner retinal activity over time.

ERG analysis at four months of age revealed that NAC treatment significantly improves absent b-wave/slow PIII amplitudes in *Bbs10*^−/−^ mice (Figure 6A). We define these as the third oscillatory peak between 55–65 ms. This was chosen because this latency is the typical timeframe when a b-wave is seen in *Bbs10*^−/−^ mice. While both water and NAC-treated *Bbs10*^−/−^ mice show decreased a- and b-wave amplitudes over time, NAC-treated mice demonstrated higher amplitudes compared to those receiving water (Welch’s t-test, p = 0.0029) (Figure 6B–6C). This indicates that NAC treatment helps to prevent the absent b-wave/slow PIII response. Furthermore, the proportional relationship between the a-wave and the negative wave amplitudes at 55–65ms, which is expected to decrease as a-wave amplitudes decline, was restored in NAC-treated mice (simple regression model, p = 0.0036) (Figure 6D). At four months, 50% of NAC-treated eyes retained b-waves, compared to only 12.5% in the water-treated group (Fisher’s exact test, p = 0.0421), highlighting the efficacy of NAC in slowing the loss of the b-wave response (Figure 7).

## Discussion

### NAC as a potential therapy for BBS10-related retinal degeneration

Our study investigates the potential therapeutic effects of NAC *in Bbs10*^−/−^ mice, a model for retinal degeneration. BBS10 is a key gene involved in cilia function, and its disruption leads to progressive photoreceptor degeneration, particularly through mechanisms like oxidative stress. NAC, an antioxidant that acts by boosting glutathione levels, has been shown to mitigate oxidative stress in various degenerative conditions, including retinal diseases, and it also has anti apoptotic properties [23,30]. Here, we demonstrate that NAC treatment improves retinal function *in Bbs10*^−/−^ mice, as evidenced by the preservation of the ERG b-wave response, which is typically absent in untreated *Bbs10*^−/−^ mice by 4 months of age. This preservation suggests that NAC may help maintain synaptic function and protect retinal neurons from degeneration.

### Transient loss-of b-wave shares similar waveforms with congenital no b-waves (nob)

While the nob ERGs are congenital, as observed in congenital stationary night blindness (CSNB), and our phenotype is transient, both exhibit similar ERG waveforms, suggesting analogous bipolar cell dysfunction [16,31,32]. In CSNB, this dysfunction is often attributed to impaired signaling between photoreceptors and ON-bipolar cells due to mutations in genes like GRM6, TRPM1, NYX, and CACNA1F [16,33]. These mutations disrupt the ability of ON-bipolar cells to respond appropriately to glutamate released from photoreceptors, resulting in the characteristic ‘no b-wave’ phenotype observed in CSNB [16].

Similarly, the loss-of-b-wave phenotype in our model may reflect transient bipolar cell dysfunction arising from an analogous disruption of bipolar cell signaling, albeit via a distinct mechanism. We hypothesize that this dysfunction is due to improper synapse structure, where presynaptic terminals from photoreceptors are mislocalized to the ONL as the photoreceptor sickens due to cilial dysfunction, while postsynaptic bipolar cell dendrites remain confined to the OPL with the cell process stretched into the ONL (Figure 4), eventually losing successful synaptic connections with the mislocalized presynaptic terminals. This structural disconnect likely contributes to the inability of bipolar cells to receive and propagate signals, leading to the observed loss-of-b-wave phenotype.

Together, these findings suggest that while CSNB and our transient loss-of-b-wave phenotype differ in their genetic underpinnings and timing, both conditions share a downstream consequence of impaired bipolar cell function. In CSNB, this arises from molecular defects disrupting signal transduction at the photoreceptor-to-bipolar cell synapse. In contrast, our model implicates structural abnormalities at the synapse, where mislocalized presynaptic terminals fail to successfully connect with postsynaptic dendrites. This comparison highlights how different mechanisms can converge on a similar functional outcome: loss of bipolar cell-driven b-wave generation.

### The absent b-wave in *Bbs10*^−/−^ mice correlates with synaptic mislocalization

The absence of the b-wave phenotype has not been previously reported in the *Bbs10*^−/−^ model, and it provides new insight into the nature of retinal degeneration in BBS10-related diseases. The b-wave, which reflects the function of bipolar cells in response to light stimuli, is typically a reliable indicator of retinal function. The loss of the b-wave in *Bbs10*^−/−^ mice suggests that retinal dysfunction in this model progresses through a stage of synaptic dysfunction rather than photoreceptor loss alone. This highlights a potential therapeutic target for BBS10-related retinal degenerations: preserving synaptic function may be as crucial as preventing photoreceptor degeneration. The retention of the b-wave in NAC-treated mice further supports this notion, demonstrating the therapeutic potential of targeting oxidative stress to preserve synaptic function and, by extension, retinal health. Of interest, mice and people with BBS have developmental cognitive anomalies which may be related to synaptic dysfunction in the brain.

### Synaptic architecture and its role in the b-wave phenotype

Our study included presynaptic terminal staining to investigate the localization of synapses in untreated *Bbs10*^−/−^ mice. The presynaptic terminals, which are critical for synaptic signaling between photoreceptors and bipolar cells, show mislocalization in untreated *Bbs10*^−/−^ mice. Interestingly, even when the ONL remains structurally consistent across different *Bbs10*^−/−^ mice, the degree of presynaptic terminal mislocalization varies, indicating that the phenotype is not due to a more severely degenerated retina. This variation in presynaptic terminal localization correlates with different ERG phenotypes, including the presence or absence of the b-wave.

Untreated *Bbs10*^−/−^ mice exhibit a mix of ERG b-wave phenotypes clustered at the 90 to 97 days-old age range, which correspond to varying degrees of presynaptic terminal mislocalization. After this time period, most eyes have a nonrecordable standard combined response ERG. In particular, mice that retain an electropositive b-wave exhibit less presynaptic terminal mislocalization compared to those with an absent b-wave. This suggests that the preservation of synaptic function, as indicated by the b-wave, is closely tied to the correct localization of presynaptic terminals. When treated with NAC, we observed a retention of the b-wave on ERG. This finding strongly suggests that NAC helps maintain proper synaptic architecture by preventing the mislocalization of presynaptic terminals, which in turn supports more efficient synaptic signaling between photoreceptors and bipolar cells.

The synaptic architecture in *Bbs10*^−/−^ mice provides a mechanistic explanation for the variation in ERG b-wave phenotypes. In retinas with an absent b-wave, presynaptic terminals labeled with PSD95 are severely mislocalized, and only limited connections with postsynaptic terminals, visualized via PKCα, are observed. This indicates a breakdown in synaptic integrity, likely preventing functional neurotransmission and explaining the absence of a b-wave in the ERG.

In contrast, *Bbs10*^−/−^ retinas that retain an electropositive b-wave exhibit fewer mislocalized presynaptic terminals and show intact connections between presynaptic PSD95 and postsynaptic PKCα-labeled bipolar cell dendritic tips, even when mislocalized to the ONL. This suggests that, while mislocalized, the synaptic architecture in these retinas remains functional to some extent, enabling neurotransmission and the generation of a detectable b-wave. These findings align with the ERG data, reinforcing the conclusion that functional synaptic connections are critical for preserving the b-wave response.

Interestingly, similar synaptic mislocalizations of PKCα in the ONL have been observed in other mouse models, such as the DHDDS K42E mouse model, which exhibits an electronegative ERG phenotype, similar to our electronegative ERG phenotype [34]. However, in contrast to the true loss-of b-wave observed in our *Bbs10*^−/−^ mice, the DHDDS model does not completely lose the b-wave, indicating that the severity of synaptic dysfunction may differ between models. In our *Bbs10*^−/−^ model, the lack of PKCα connection to PSD95 terminals in the ONL in retinas with a loss-of b-wave further supports the notion that synaptic integrity is crucial for the generation of a recordable b-wave.

### Structural vs. Functional Preservation: ONL and ERG b-wave findings

A key observation in this study is the preservation of the ERG b-wave response in NAC-treated *Bbs10*^−/−^ mice and not the a-wave, despite the apparent preservation of the ONL in OCT images. These findings raise an important distinction between structural and functional preservation in the retina. While OCT imaging shows that the ONL appears relatively intact in treated mice, it is important to note that this structural preservation does not always correlate with functional integrity.

The b-wave functionality, which reflects the activity of bipolar cells, may be preserved even in the presence of no retinal structural differences. This highlights the importance of considering both functional and structural outcomes when evaluating the effectiveness of therapeutic interventions. The lack of correlation between INL thickness and b-wave retention underscores the complexity of retinal degeneration, where structural changes do not always predict functional outcomes. This observation suggests that the retinal degeneration in *Bbs10*^−/−^ mice involves disruptions at the level of synaptic transmission, rather than merely the loss of photoreceptor cells. Thus, the preserved b-wave in NAC-treated *Bbs10*^−/−^ mice suggests that NAC not only mitigates oxidative stress but also helps preserve synaptic function, providing a functional benefit even in the face of structural challenges.

### NAC’s role in enhancing neurotransmission and synaptic function

In addition to its antioxidant properties, NAC may also support retinal function by enhancing neurotransmission, particularly in the rod-bipolar cell synapse. Rod-bipolar transmission is heavily dependent on the release of glutamate, which acts as the key excitatory neurotransmitter at this synapse [35]. In neurological diseases, oxidative stress can impair glutamate receptor function and disrupt the synaptic machinery, ultimately leading to reduced synaptic efficiency, functional decline, and neuronal cell death [36,37]. Since neurons are the primary cell in the retina, this is a plausible mechanism in retinal degenerative diseases like BBS10. Given NAC’s ability to modulate glutathione levels and reduce oxidative damage, it is plausible that NAC may help stabilize glutamate receptors and other synaptic proteins, thereby improving synaptic signaling. This could explain the higher b-wave amplitudes observed in the NAC-treated *Bbs10*^−/−^ mice. By maintaining proper glutamatergic signaling at the rod-bipolar synapse, NAC may help preserve synaptic transmission, even in the context of ongoing retinal degeneration. As a result, the improved b-wave amplitude in NAC-treated mice could reflect not only the preservation of bipolar cell function but also enhanced neurotransmission at the synaptic level, helping to sustain the overall retinal signaling pathway.

### Potential for long-term effects and synaptic plasticity in *Bbs10*^−/−^ mice

One of the critical questions raised by our study is whether the observed improvements in retinal function, particularly the preservation of the ERG b-wave, are sustainable over the long term. While we have shown significant therapeutic benefits at the 4-month mark, it is essential to consider the potential for long-term effects, especially with chronic antioxidant treatment. Oxidative stress and its associated damage can cause irreversible changes in retinal structure and function, and NAC may help slow, but not entirely halt, the progression of retinal degeneration.

Additionally, NAC’s ability to preserve synaptic function in the retina may have broader implications for synaptic plasticity. In the context of retinal degeneration, plasticity refers to the retina’s ability to adapt and reorganize its synaptic connections in response to damage or dysfunction. Although retinal synapses are relatively stable in adulthood, evidence suggests that in some retinal degenerative diseases, such as retinitis pigmentosa, synaptic reorganization can occur in response to photoreceptor loss. NAC’s potential to protect synaptic function could enable the retina to retain some degree of synaptic plasticity, allowing for more efficient signal transmission between remaining retinal neurons. This, in turn, could help preserve visual function for a longer period, even as photoreceptor loss continues. Long-term studies, including ERG measurements and histological analyses, will be necessary to assess the durability of these improvements and determine whether NAC can maintain retinal plasticity over time.

### Weaknesses of this study

While this study examines changes in ERG b-wave evolution over four months, a longer time course for NAC treatment could also provide a clearer picture of its potential long-term efficacy. Additionally, further mechanistic insights into how NAC interacts with the underlying disease processes, how NAC specifically impacts synaptic connectivity, and molecular or cellular changes are being driven by NAC treatment are needed.

## Conclusions and Future Directions

In conclusion, our data provide compelling evidence that NAC treatment can preserve retinal function in *Bbs10*^−/−^ mice, likely by mitigating oxidative stress and preserving synaptic integrity. The retention of the ERG b-wave in treated mice suggests that NAC may help maintain the function of retinal neurons, particularly bipolar cells, which are essential for synaptic transmission and visual processing. The molecular analysis of presynaptic terminals further supports the idea that NAC helps preserve synaptic function by preventing mislocalization of synaptic components. While our findings highlight the potential of NAC as a therapeutic approach for BBS10-related retinal degeneration, further studies are needed to explore the underlying mechanisms, including the role of mitochondrial function, protein misfolding, and cell death pathways. Additionally, future work should investigate the long-term effects of NAC treatment and its impact on retinal plasticity and visual function, as well as the potential for combination therapies that address both oxidative stress and other disease pathways. Ultimately, NAC’s antioxidant properties may offer a promising strategy for slowing or halting the progression of retinal degenerations caused by ciliary dysfunction.

## Supplementary Material

JEN-25-108_Supplemental File

## Figures and Tables

**Table 1.** Primers for *Bbs10* genotyping.

Primer	Ratio (%)	Sequence 5′ to 3′
Bbs10KO-WT-Fr	20	CCCATGGTAAGTGGTCAATCAG
Bbs10KO-MT-Fr	45	TCAATGTATCTTATCATGTCTG
Bbs10KO-WT-Rv	35	TGGTCTGGTGGACTCAATGGAC

## Highlights

- Bardet-Biedl Syndrome Type 10 retinal degeneration goes through a loss-of-b-wave synapse phenotype on electroretinogram.
- N-Acetylcysteine slows this phase of retinal degeneration.
