# Supplementary figures and images for: N-Acetylcysteine Ameliorates Loss of the Electroretinogram b-wave in a Bardet-Biedl Syndrome Type 10 Mouse Model
Source: J Exp Neurol. Author manuscript; Available in PMC 2025 Sep 25. (PMC12459551; doi:10.33696/Neurol.6.108)

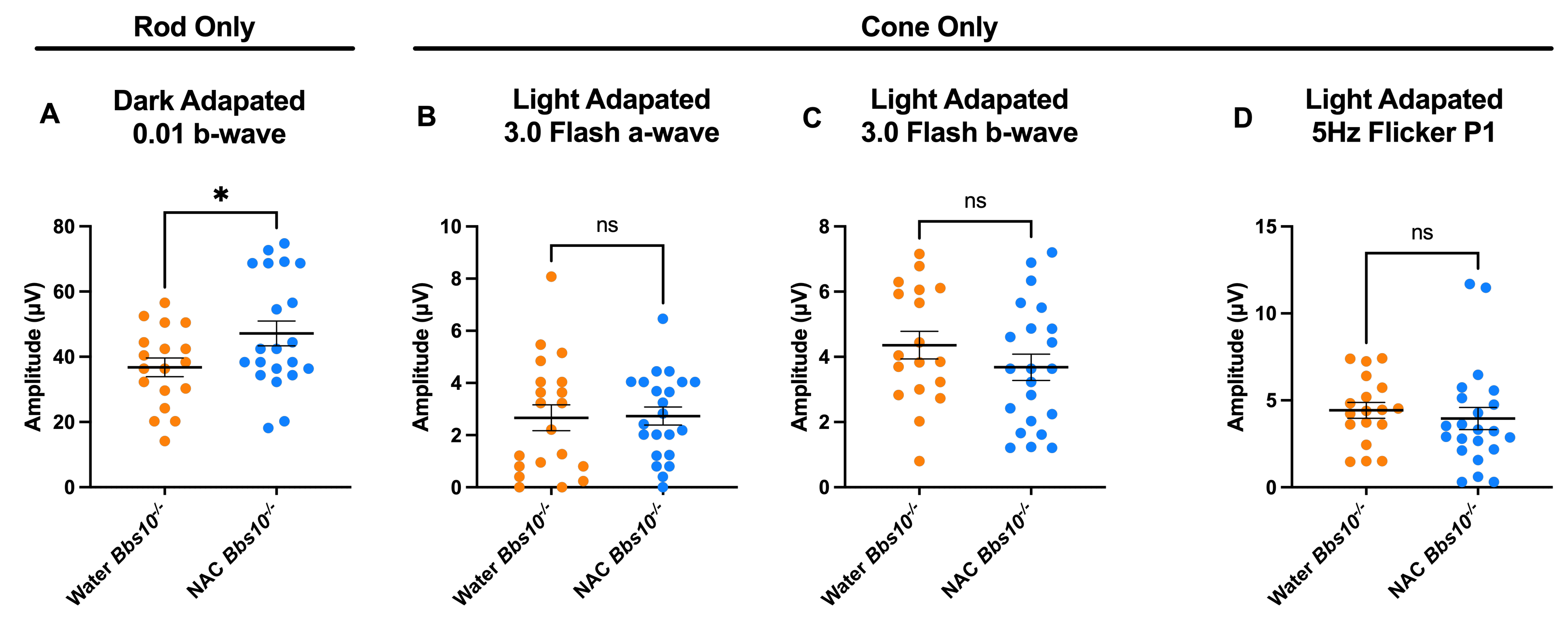

Supplement: JEN-25-108_Supplemental File [file NIHMS2101076-supplement-JEN-25-108_Supplemental_File.zip › Supplemental Figure 1.tiff]
